# Historical introgression from commercial meat-type ducks constitutes the common genetic basis of white plumage in egg-type duck breeds

**DOI:** 10.1016/j.psj.2026.107827

**Published:** 2026-09-22

**Authors:** Chunyuan Zhang, Zhen Chen, Pan Li, Panxue Wu, Xuge Wang, Chungang Feng, Yiqiang Zhao, Lizhi Lu, Li Chen

**Affiliations:** a Institute of Animal Husbandry and Veterinary Medicine, Xianghu Laboratory, Zhejiang Academy of Agricultural Sciences, Hangzhou, 311231, China; b State Key Laboratory of Animal Biotech Breeding, College of Biological Sciences, China Agricultural University, Beijing, 100193, China; c College of Animal Science and Technology, Nanjing Agricultural University, Nanjing, 210095, China; d Zhejiang-Ukraine Joint Laboratory for Poultry Germplasm Resources Conservation, Exploitation and Utilization, Zhejiang Academy of Agricultural Sciences, Hangzhou, 311231, China

**Keywords:** Shaoxing duck, White plumage, *MITF*, ∼6.6kb insertion mutation, Introgression

## Abstract

White plumage is an important economic trait in duck, but the genetic mechanism underlying white plumage in egg-type ducks remains unclear. In this study, white plumage individuals were obtained from a naturally segregating population of the grey plumage line of Shaoxing ducks. Cross families were constructed through reciprocal mating between white and grey-plumage lines for genetic mapping. Genome-wide association analysis mapped the white plumage trait to chromosome 13 and identified *MITF* as the sole candidate gene. Structural variation detection revealed an approximately 6.6 kb Gypsy insertion between exon 1M and exon 2 of *MITF* (the variant underlying white plumage in meat-type ducks), homozygous in all white individuals. Across 1,192 individuals from 38 populations, this insertion was fixed in all commercial meat-type ducks and in the indigenous white-plumage lines of Jinding, Putian black and Shaoxing ducks (but absent in Liancheng white duck), establishing the *MITF* insertion as the common genetic basis for white plumage across these lines. Population genetic structure analysis showed that each indigenous white-plumage line clustered closely with its corresponding colored-plumage population, with no detectable signals of large-scale hybridization from commercial meat-type ducks. Although indigenous white-plumage lines have considerably shorter breeding history than commercial meat-type ducks, the positive selection signature at the *MITF* locus in these lines is highly similar to that in the latter, with both sharing a nearly homozygous haplotype block of approximately 31.6 kb located in a genomic interval of markedly reduced recombination rate. Haplotype network analysis placed the insertion-carrying haplotypes of indigenous white-plumage lines in a haplogroup shared with commercial meat-type ducks. Collectively, these results support the interpretation that the *MITF* insertion was historically introgressed from commercial meat-type ducks, persisted at low frequency in indigenous populations, and was subsequently driven to rapid fixation under recent market-driven selection, thereby generating selection signatures highly consistent with those in commercial meat ducks within a short period.

## Introduction

Plumage coloration represents a pivotal economic trait in domestic ducks, exerting profound influences on carcass appearance, consumer preference, and the commercial value of down-based products. Domestic ducks were primarily domesticated from the Mallard (*Anas platyrhynchos*), during which the lineages leading to present-day commercial meat-type ducks and local breeds rapidly diverged (Guo, et al., 2020; Li, et al., 2010; Zhang, et al., 2018; Zhang, et al., 2025). Historically, white plumage has been preferentially selected in commercial meat-type ducks, particularly in Peking duck, driven by its favorable effects on carcass appearance and consumer acceptance. In contrast, traditional indigenous ducks have predominantly retained colored plumage patterns characteristic of their wild ancestor. This divergent selection history has resulted in white plumage genetics being extensively studied in meat-type ducks, while the genetic basis of white plumage in indigenous ducks remains largely unexplored.

Khan et al. reviewed the genetic mechanisms underlying duck plumage coloration, with particular emphasis on the melanogenesis pathway and its core regulatory genes (*MITF, MC1R, TYR, TYRP1, DCT, SOX10, KIT, EDNRB2*, and *MLANA*), as identified through GWAS, RNA sequencing, and whole-genome resequencing (Khan, et al., 2025). In meat-type ducks, particularly Peking duck, the genetic basis of white plumage has been well characterized through studies of the *MITF* gene, which encodes a transcription factor critical for the specification and survival of neural crest-derived melanocytes. Using a cross population between Mallard and Peking duck, Zhou et al. first identified an approximately 6.6 kb insertion within the *MITF* gene as a candidate causal variant for white plumage in Peking duck, likely by disrupting the expression of the melanocyte-specific MITF-M transcript (Zhou, et al., 2018). Wang et al. subsequently refined this finding through pan-genome assembly, demonstrating that a 6,634 bp Gypsy insertion between exon 1M and exon 2 abolishes normal MITF-M transcript expression and instead drives production of a novel transcript that fails to activate the downstream melanogenesis pathway, resulting in white plumage (Wang, et al., 2024a). Consistent with this, melanin is almost undetectable in the feather follicles of Peking duck (Wang, et al., 2024b). Using RACE technology, Wang et al. and Lin et al. independently identified structurally similar MITF-M transcript variants overlapping with intron 1 from skin tissue of Peking duck and feather bulbs of white-plumage Putian black duck, respectively (Lin, et al., 2024; Wang, et al., 2024a). Consistent with this mechanism, knockout of the MITF-M isoform in mice results in the absence of mature neural crest-derived melanocytes in the skin, producing a white coat phenotype (Flesher, et al., 2020). Furthermore, the *MITF* insertion has been shown to exert epistatic effects on other plumage-color-related genes, with the depletion of mature melanocytes in the skin proposed as the mechanistic basis of this epistasis (Gong, et al., 2010; Wang, et al., 2025). In addition, SNP mutations in the MITF-M promoter and differential CpG methylation at the *MITF* locus have been implicated in regulating the transition between black and white plumage phenotypes in Putian black duck (Lin, et al., 2019; Lin, et al., 2024).

The possibility of distinct genetic mechanisms is supported by the Liancheng white duck, a small-size meat-type breed distinguished by its white plumage but black beak and web, a unique phenotype that sets it apart from Peking duck, which displays white plumage with yellow beak and web. High-quality genome assembly revealed that an incompletely dominant mutation in *PMEL* that impairs melanosome transport is a key determinant of this distinctive plumage phenotype (Wang, et al., 2025). Consistently, unlike Peking duck which has almost no detectable melanin in its feathers, Liancheng white duck retains a small but measurable amount of melanin, though almost no obvious melanin distribution is observed in the feather follicles of either breed (Wang, et al., 2024b). Beyond white plumage, *EDNRB2* has been identified as a candidate gene for the black-and-white spot plumage pattern in ducks, with a mutation in its coding sequence associated with the presence of spots (Li, et al., 2015), and SNPs in its regulatory region shown to modulate spot size in a dose-dependent manner (Xi, et al., 2021). These examples demonstrate that different duck breeds can achieve white plumage through distinct genetic mechanisms, highlighting the importance of investigating the genetic basis of white plumage in each lineage independently.

In recent years, several indigenous ducks in China have initiated the development of white-plumage lines, including white-plumage lines of Jinding duck, Putian black duck, and Shaoxing duck. These populations provide a unique opportunity to investigate the genetic architecture of white plumage in indigenous ducks and to determine whether this trait evolved independently or shares a common genetic basis with meat-type ducks. In this study, white-plumage Shaoxing ducks were derived from a grey-plumage line that had itself been naturally segregated from the mallard-plumage Shaoxing duck population, and reciprocal cross families were constructed between white-plumage and grey-plumage lines. By integrating our dataset with publicly available genomic resources, we aimed to: (1) identify the genetic variant underlying white plumage in the Shaoxing duck; (2) determine whether this variant is shared across indigenous white-plumage lines; and (3) investigate the evolutionary origin and selection dynamics of white plumage in indigenous duck populations.

## Materials and methods

### Ethics statement

Experimental protocols complied with the regulations and guidelines of the Animal Care and Use committee of the Zhejiang Academy of Agricultural Sciences.

### Animals and cross-family construction

Grey-plumage individuals were selected from a naturally segregating population derived from the mallard-plumage Shaoxing duck, and white-plumage individuals were subsequently selected from a naturally segregating population derived from the grey-plumage line. Using white-plumage and grey-plumage lines as parental stocks, four cross combinations were established (white♂ × white♀, white♂ × grey♀, grey♂ × white♀, and grey♂ × grey♀), with 10 white-plumage sires, 10 grey-plumage sires, 13 white-plumage dams, and 64 grey-plumage dams, yielding a total of 129 families and 512 F1 offspring (Table S1). Offspring were assigned to white-plumage or grey-plumage phenotypic classes based on visual inspection. Segregation ratios were tested by χ² goodness-of-fit within families in which the grey parent produced at least one white offspring. All animals were housed within the same facility and fed a standard diet containing 17.5% crude protein and 11.5 MJ/kg metabolizable energy. Brooding temperature was maintained at 32–35°C under natural lighting. The two lines were reared in separate floor pens with ad libitum access to feed and water, and each pen was equipped with an independent pool to accommodate the aquatic behavioral needs of the birds.

### Samples and resequencing

A total of 243 Shaoxing duck individuals were selected for whole-genome resequencing, comprising 90 grey-plumage and 73 white-plumage individuals from the cross families, and 80 mallard-plumage individuals. Peripheral blood was collected from the wing vein of each individual into EDTA-containing anticoagulant tubes and stored at 4°C. DNA quantity was assessed using the Quant-iT PicoGreen dsDNA Assay Kit, and qualified samples were used for library construction with the Illumina TruSeq Nano DNA LT kit. Whole-genome resequencing was performed on the Illumina NovaSeq X Plus platform with 150 bp paired-end reads at a sequencing depth of 10 × .

Publicly available whole-genome resequencing data were downloaded from NCBI under the following BioProject accessions: PRJNA972858, PRJNA658213, PRJNA409830, PRJNA419832, PRJNA961294, PRJNA686828, PRJNA401972, PRJCA011446, PRJNA599025, PRJNA781414, PRJNA1143704, PRJNA1088559, PRJNA1145904, PRJNA554956, PRJNA844232, PRJNA668048, PRJNA896757, PRJNA1000221, PRJNA888455, and PRJNA450892. Additional data were downloaded from the Genome Sequence Archive (GSA; http://gsa.big.ac.cn/) under accession CRA001690, and Jiaji duck (*Cairina moschata*) resequencing data were obtained from BioProject PRJNA1178879. For the population-level analyses, samples were assigned to groups on the basis of plumage color, *MITF* insertion genotype and genetic background. Commercial comprises Peking, Cherry Valley, Grimaud Frères and Maple Leaf ducks. Indigenous White comprises the white-plumage lines of Jinding, Putian black and Shaoxing ducks, all three of which are egg-type breeds. Indigenous Colored Counterparts comprises the three populations from which these lines were derived, namely Jinding duck, Putian black duck and the grey-plumage line of Shaoxing duck. Indigenous Colored comprises the remaining colored indigenous populations. Liancheng white duck, whose white plumage has been attributed to *PMEL* (Wang, et al., 2022; Wang, et al., 2025), is treated separately, as are Mallard, the principal ancestor of domestic ducks, and Spot-billed duck and Jiaji duck, used as outgroups. Plumage color is recorded for individual birds only in Jinding white, Putian white and Putian black ducks; for the remaining public samples it was assigned from the breed standard, the original studies not reporting the color of individual samples. Group assignments for all samples are listed in Table S3.

### Sequence alignment and variant calling

Raw reads were trimmed to remove adapter contamination using AdapterRemoval v2 (Schubert, et al., 2016). Clean reads were aligned to the IASCAAS_PekinDuck_T2T (GCF_047663525.1) reference genome using BWA v0.7.17 (Li, 2013) with the mem algorithm under default parameters. Alignment files were sorted, converted to BAM format, and processed for PCR duplicate marking using Picard v1.107 (MarkDuplicates). All samples were jointly genotyped using GATK UnifiedGenotyper (stand_call_conf = 30, stand_emit_conf = 10) (DePristo, et al., 2011). At the sample level, individuals with a sequencing depth ≥ 5 × and an individual missing rate < 10% were retained, leaving 1,192 individuals for downstream analyses. At the variant level, sites with a missing rate ≥ 10% or a minor allele frequency (MAF) < 0.05 were excluded, and 10,051,665 autosomal SNPs were retained. Structural variants were called across all samples using Delly (v1.5.0) (Rausch, et al., 2012). A structural variant on chromosome 13 at positions 5,186,889–5,193,521 (CIPOS = −8, 8) was identified as the target mutation. Because the reference assembly derives from Peking duck, which carries the element, the variant is returned as a deletion relative to the assembly (0/0 denoting homozygous presence of the element); it is referred to throughout as an insertion, following the ancestral state and the terminology of previous studies (Wang, et al., 2024a; Zhou, et al., 2018). Low-quality genotype sites and samples genotyped as 0/0 in the colored-plumage group were set as missing. The quality-controlled SNPs and structural variants were then merged and subjected to imputation and phasing using BEAGLE (v5.5) (Browning, et al., 2021; Browning, et al., 2018). Haplotype networks were constructed and visualized using HapNetworkView (v1.0) (Chi, et al., 2025). The sequence of the *MITF* gene was extracted from the reference genome, and LTR Finder (v1.0.7) (Xu and Wang, 2007) was used to detect the long terminal repeats (LTRs) flanking both sides of the Gypsy insertion.

### GWAS and candidate gene identification

A genome-wide association study (GWAS) was conducted on 243 Shaoxing ducks sequenced in this study, with plumage color as the phenotype. Association was tested by the Wald test in the univariate linear mixed model implemented in GEMMA (v0.98.3) (Zhou and Stephens, 2012), with a genomic relatedness matrix fitted as a random effect to account for relatedness and population structure. Genome-wide significance was set by Bonferroni correction for the number of SNPs tested (P < 4.97 × 10⁻⁹; −log₁₀P = 8.30), and the genomic inflation factor (λ) was estimated as the ratio of the median observed to the median expected χ² statistic. To refine the candidate region, individuals from the cross families were assigned genotypes GG, Gg or gg at the plumage-color locus on the basis of pedigree records and Mendelian segregation (Table S2), and significant SNPs were retained only if their genotypes were consistent with gg in all white-plumage individuals and with Gg in the 26 grey-plumage individuals identified as carriers. A conditional GWAS was performed by adding the *MITF* insertion dosage as an additional covariate, using the same significance threshold.

### Population structure, haplotype block definition and positive selection analysis

Genetic ancestry components of ducks (*Anas platyrhynchos*) were estimated using ADMIXTURE (v1.3.0) (Alexander, et al., 2009), with the number of ancestral populations K ranging from 2 to 8. Genetic distances among populations were assessed using genome-wide F_ST_, based on which a neighbor-joining (NJ) phylogenetic tree was constructed at the population level. Per-site Weir and Cockerham's F_ST_ and nucleotide diversity (θπ), and Tajima's D in non-overlapping 3 kb windows, were calculated using VCFtools (v0.1.16) (Danecek, et al., 2011). The haplotype block surrounding the insertion was delimited from the genotype heatmap of *MITF* variants (Fig. 3A) as chr13:5,177,535–5,209,172 (31,638 bp). Signatures of positive selection were detected by scanning extended haplotype homozygosity (EHH) using selscan (v2.1.2) (Szpiech and Hernandez, 2014). The effective population size estimated in (Feng, et al., 2021) was used as an input parameter for Pyrho (v0.2.1) (Kamm, et al., 2016; Spence and Song, 2019) to infer the genomic recombination rate.

### Introgression analysis

F3 statistics were calculated with qp3Pop (v701) from the ADMIXTOOLS package (Patterson, et al., 2012; Reich, et al., 2009) for all possible three-population combinations to test whether the target population is the product of large-scale admixture (e.g., hybridization events) between two reference populations. Using the Spot-billed Duck (*Anas poecilorhyncha*) as the outgroup, D statistics (ABBA-BABA test) were computed with qpDstat (v1152) for all possible population combinations to detect signals of gene flow among populations (Durand, et al., 2011). Standard errors were obtained by block jackknife and |Z| > 3 was taken as significant. The f4-ratio, computed with qpF4Ratio (v400), was used to compare the extent of gene flow from commercial meat-type ducks between each white-plumage line and its corresponding colored-plumage population (Patterson, et al., 2012). The Spot-billed duck was fixed as outgroup O and the Mallard as reference population A. All combinations were then enumerated in which source population B was one of the four commercial meat-type duck populations (Peking, Cherry Valley, Grimaud Frères and Maple Leaf ducks), reference population C was one of the indigenous colored-plumage populations, and test population X was one of the six target populations, namely the white-plumage lines of Jinding, Putian black and Shaoxing ducks together with their three corresponding colored-plumage populations. Combinations were retained only when α fell within the interval (0, 1) and |Z| > 3, and those failing either criterion are not shown. Because the comparison of interest is between the two lines of a breed, only those reference populations C that were retained for all three breed pairs were carried forward, so that all six target populations were evaluated under a common set of (B, C) configurations. Three populations satisfied this requirement (Gaoyou duck, Weishan Ma duck and Wendeng black duck) yielding 68 estimates, of which 32 form complete pairs in which both lines of a breed gave a significant estimate.

## Results

### A ∼6.6 kb MITF insertion underlies white plumage in Shaoxing Duck

Reciprocal cross families were constructed using grey-plumage and white-plumage lines as parental stocks, yielding a total of 129 families and 512 offspring (Table S1). The white♂ × white♀ combination produced only white offspring (52 of 52, 12 families), and the grey♂ × white♀ combination only grey offspring (31 of 31, 9 families). Because the grey-plumage line is itself segregating, the two remaining combinations are informative only in families whose grey parent proved to be a carrier. Within the 18 white♂ × grey♀ families that produced white offspring, the ratio was 41 white to 47 grey, as expected for a testcross of a heterozygote (χ² = 0.41, P = 0.52). Plumage color was independent of sex in the two combinations in which both phenotypes occurred (P = 0.50 and P = 0.75). Grey plumage is therefore completely dominant over white, and white plumage is recessive and autosomal. Although the white-plumage line was derived along a mallard-to-grey-to-white path, white plumage was epistatic to grey in the cross families examined here, and the genetic basis of grey plumage was not investigated further in this study. To further map the candidate gene underlying white plumage, 90 grey-plumage and 73 white-plumage individuals were selected from the cross families, along with 80 mallard-plumage individuals, for whole-genome resequencing (Table S2). GWAS identified a single strong association peak on chromosome 13, where 245 SNPs between 1.87 and 6.06 Mb exceeded the significance threshold; elsewhere, only an isolated SNP on chromosome 5 did so (Fig. 1A). The genomic inflation factor was 0.97, and observed P values departed from the null expectation only in the extreme tail (Fig. 1A, inset), indicating that relatedness and population structure were adequately controlled. This 4.19-Mb region contains 14 candidate genes, representing the major quantitative trait locus (QTL) for white plumage (Fig. 1B). Of the 245 significant SNPs, 8 showed genotypes consistent with the expected segregation pattern in the cross families (Table S2), all mapping to the intronic region between exon 1M and exon 2 of *MITF* (Fig. 1C). Delly identified a ∼6.6 kb structural variant within the intronic region between exon 1M and exon 2 of *MITF* (chr13:5,186,889–5,193,521; CIPOS = −8, 8) flanked by LTR sequences, confirming it as a Gypsy insertion (Fig. S1). A conditional GWAS incorporating the insertion dosage as an additional covariate abolished association signals on chromosome 13, no SNP reaching P < 4.97 × 10⁻⁹ (Fig. 1D), indicating that this structural variant statistically accounts for the entirety of the GWAS signal at the *MITF* locus. Notably, this insertion occupies the same intronic position as the ∼6.6 kb Gypsy insertion previously reported in Peking duck, which disrupts MITF-M transcription and blocks melanogenesis (Wang, et al., 2024a), strongly suggesting that white plumage in the Shaoxing duck is most likely caused by the same molecular mechanism.

**Fig. 1:**
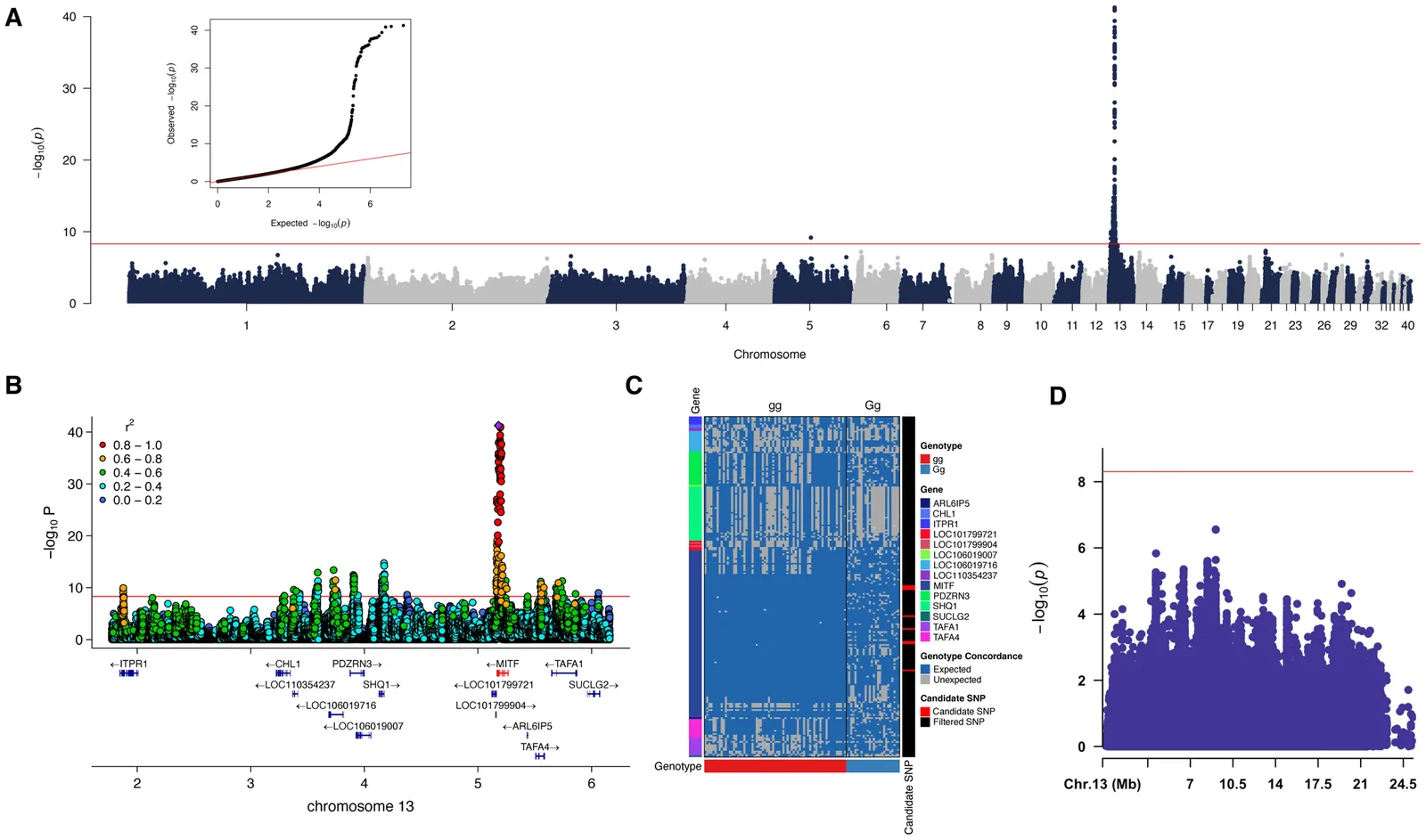
Identification of candidate genes for white plumage via GWAS. (A) Manhattan plot of the GWAS for white plumage in 243 Shaoxing ducks; the horizontal line marks the Bonferroni-corrected significance threshold (−log₁₀P = 8.30). The inset is the quantile–quantile plot of observed against expected −log₁₀(P) for all SNPs tested, with the red line indicating the expectation under the null hypothesis. (B) Zoomed-in view of the candidate region. (C) Genotyping of variants within the candidate region and screening of candidate genes. (D) Conditional GWAS with the insertion dosage as a covariate.

### Widespread fixation of the MITF ∼6.6 kb insertion in white-plumage ducks and its low-frequency occurrence in colored-plumage populations

To determine whether the insertion mutation underlying white plumage is shared among other indigenous duck breeds, we retrieved whole-genome resequencing data from 35 populations comprising 949 individuals from public databases (Table S3). Together with the three Shaoxing duck populations sequenced in this study, this gave 1,192 individuals from 38 populations. These included commercial meat-type ducks (Peking ducks, Cherry Valley, Grimaud Frères, and Maple Leaf ducks), Jinding ducks, Putian black ducks and their white-plumage lines, Liancheng white ducks, multiple colored-plumage indigenous breeds, Mallards (*Anas platyrhynchos*, n = 107), Spot-billed ducks (*Anas poecilorhyncha*, n = 50), and Jiaji ducks (*Cairina moschata*, n = 30). All commercial meat-type ducks were homozygous for the *MITF* ∼6.6 kb insertion, consistent with its reported role in white plumage in this group (Wang, et al., 2024a). Extending the survey to indigenous breeds, the white-plumage lines of Jinding, Putian black, and Shaoxing ducks were likewise homozygous for the insertion, establishing it as the common genetic basis for white plumage across these three lines. In contrast, the insertion was absent in Liancheng white duck, consistent with the previously reported role of *PMEL*, rather than *MITF*, in determining its white plumage (Wang, et al., 2025). In colored-plumage populations, the insertion was detected at low frequency, including in Wendeng black ducks (11.7%), Weishan ducks (5.0%), Matahu ducks (3.3%), Youxian ducks (2.2%), and Jinding ducks (1.4%), among other breeds; notably, only a single heterozygous individual was identified among 107 mallards, and no carriers were detected in Spot-billed ducks (Fig. S2). The insertion frequency was higher in the grey-plumage Shaoxing duck (27.2%) and Putian black duck (31.1%); both samples were drawn from cross populations, the former from the cross families of this study and the latter from an F2 population (Yang, et al., 2025), and these frequencies therefore reflect the respective cross designs.

### Indigenous white-plumage lines are most closely related to their corresponding colored-plumage populations

As noted above, the Shaoxing duck white-plumage line was selected directly from a naturally segregating grey-plumage population. To determine whether the white-plumage lines of Jinding duck and Putian black duck were similarly developed without large-scale hybridization of commercial breed genomic background, we conducted population genetic structure analysis. ADMIXTURE analysis showed that each indigenous white-plumage line shared essentially the same ancestry profile as its colored-plumage counterpart, with no shift toward the ancestry component that predominates in commercial meat-type ducks (Fig. 2A). At K = 2, where this component reached its highest values, it accounted for 6.78 ± 1.59% in Putian black duck (n = 74) and 6.48 ± 1.82% in the Putian white line (n = 39), 4.24 ± 1.58% and 4.24 ± 0.60% in Jinding duck and its white line (n = 37 and 18), and 0.32 ± 0.65% and 0.11 ± 0.53% in the grey and white lines of Shaoxing duck (n = 90 and 73). Across K = 2 to K = 8, the two lines of a given breed never differed by more than 0.5 percentage points, and no white-plumage line carried a consistently larger share of this component than its colored-plumage counterpart. Neighbor-joining trees constructed from genome-wide F_ST_ matrices likewise showed that the Shaoxing duck white-plumage line and the Putian duck white-plumage line each clustered with their respective colored-plumage populations (Fig. 2B); although the Jinding white-plumage line first clustered with Matahu duck before joining Jinding duck, it remained most closely related to Jinding duck rather than grouping with commercial meat-type ducks. F3 statistics detected no evidence of admixture from commercial meat-type ducks during white-plumage line development (Table S4), providing no support for active hybridization with Peking duck as part of the breeding process. Genome-wide mean F_ST_ between the white and colored plumage lines of the same breed was low (Jinding duck: 0.035; Putian duck: 0.038; Shaoxing duck: 0.023), well below the differentiation between each white-plumage line and Peking duck (0.151, 0.131 and 0.124, respectively). Against this low genome-wide background, F_ST_ along chromosome 13 rose sharply and locally at the interval encompassing *MITF*, reaching 0.98 between Jinding duck and its white-plumage line (Fig. 2C). Together with the ancestry, phylogenetic and F3 analyses, these results indicate that each indigenous white-plumage line was selected from within its own breed rather than developed by crossing with commercial meat-type ducks.

**Fig. 2:**
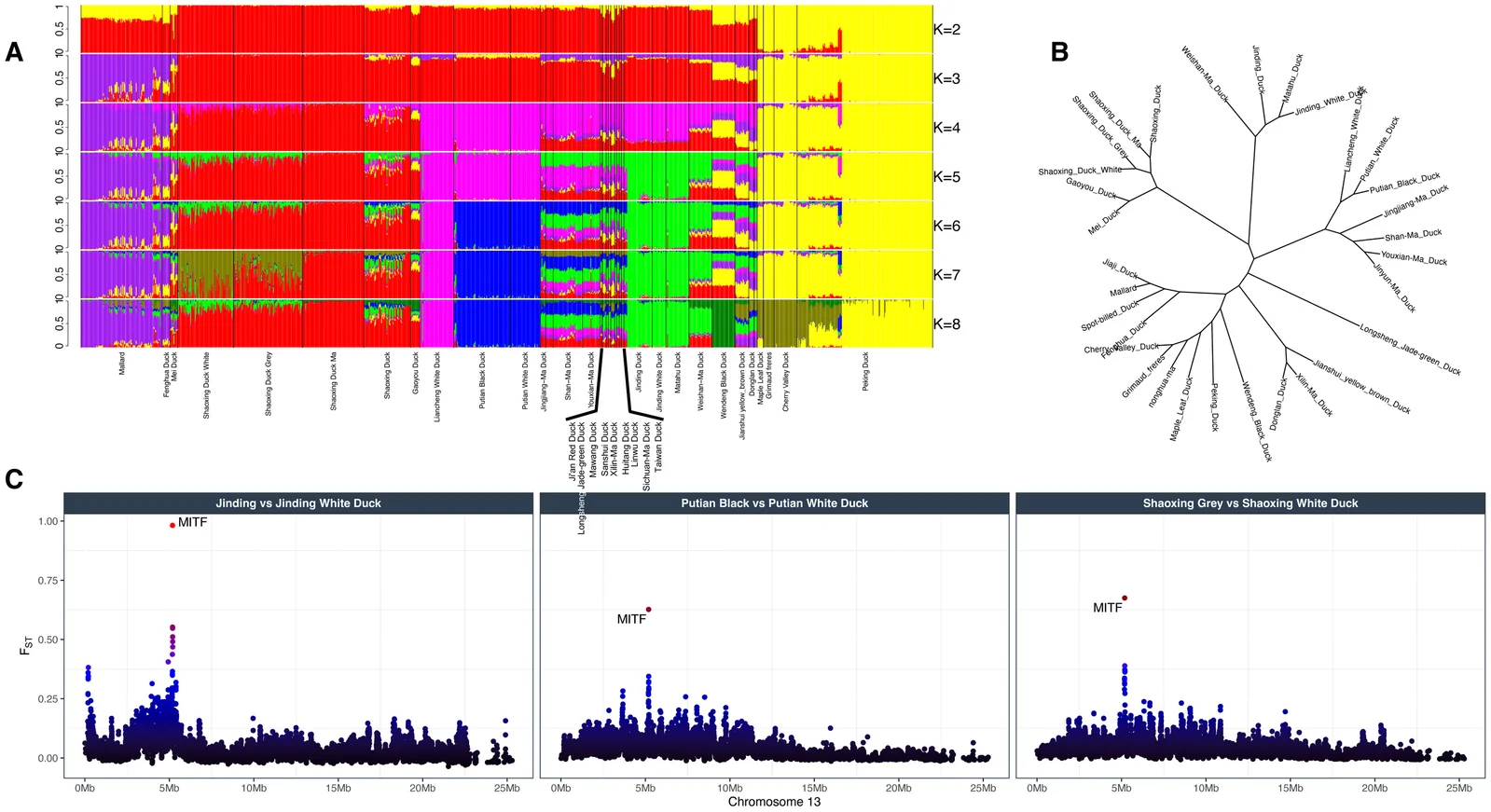
Genetic structure of white-plumage indigenous lines and their corresponding colored-plumage populations. (A) Admixture analysis of the *Anas platyrhynchos* samples (K=2-8). (B) Neighbor-joining (NJ) phylogenetic tree constructed based on the genome-wide F_ST_ matrix. (C) Per-site F_ST_ values along chromosome 13 between indigenous white-plumage duck lines and their corresponding colored-plumage populations.

### A Shared ∼31.6 kb haplotype block at MITF under positive selection across meat-type and indigenous white-plumage ducks

Although the indigenous white-plumage lines were genetically closest to their colored-plumage counterparts across the genome (Fig. 2), the pattern at the *MITF* locus was reversed. The genotype heatmap showed that the three white-plumage lines shared with commercial meat-type ducks a nearly homozygous haplotype block of approximately 31.6 kb (chr13:5,177,535–5,209,172) surrounding the insertion site and encompassing exons 1 through 6 of *MITF*, a block not shared with their colored-plumage counterparts (Fig. 3A). EHH analysis centered on the insertion site detected substantially extended homozygosity on both flanks in commercial meat-type ducks and three indigenous white-plumage lines, consistent with strong positive selection as the driving force underlying block formation (Fig. S4). In the analyses that follow, the three indigenous white-plumage lines were combined as Indigenous White and the three populations from which they were derived as Indigenous Colored Counterparts, the latter comprising Jinding duck, Putian black duck and the grey-plumage line of Shaoxing duck. Within the block, nucleotide diversity (θπ) fell to near zero in the indigenous white-plumage lines, as in Peking duck, but remained high in their colored-plumage counterparts and in Mallard (Fig. 3B), and the same reduction was observed in each of the three white-plumage lines (Fig. S3A), indicating near-complete selective sweeps. Tajima's D within the block was negative in the Indigenous White duck and positive in Peking duck (Fig. 3C). Among the individual lines, it was negative in the Jinding and Shaoxing white-plumage lines and positive in the Putian white-plumage line, in which the block was nearly monomorphic (Fig. S3B). These negative values suggest that the sweep in the Indigenous White duck is more recent than that in Peking duck, with low-frequency variants having since accumulated on the fixed haplotype. F_ST_ analysis revealed that pairwise F_ST_ between Indigenous White duck and Peking duck approached zero within the block, whereas F_ST_ between Indigenous White and Indigenous Colored Counterparts neared 1 (Fig. 3D, Fig. S6), further demonstrating that white-plumage populations of distinct breed origins share a nearly identical haplotype background at this locus. The block coincides with an interval of markedly reduced recombination rate in the white-plumage lines, in mallard and in the indigenous colored-plumage populations alike (Fig. 3E). Reduced recombination limited the erosion of haplotypes carrying the insertion, and together with directional selection for white plumage accounts for the extent and homogeneity of the block. Although the three indigenous white-plumage lines each originated from distinct indigenous breeds and have undergone relatively brief periods of selective breeding, the selective sweep signal at the *MITF* locus is nearly indistinguishable from that observed in commercial meat-type ducks, suggesting that the insertion allele most likely shares a common genetic origin across these populations.

**Fig. 3:**
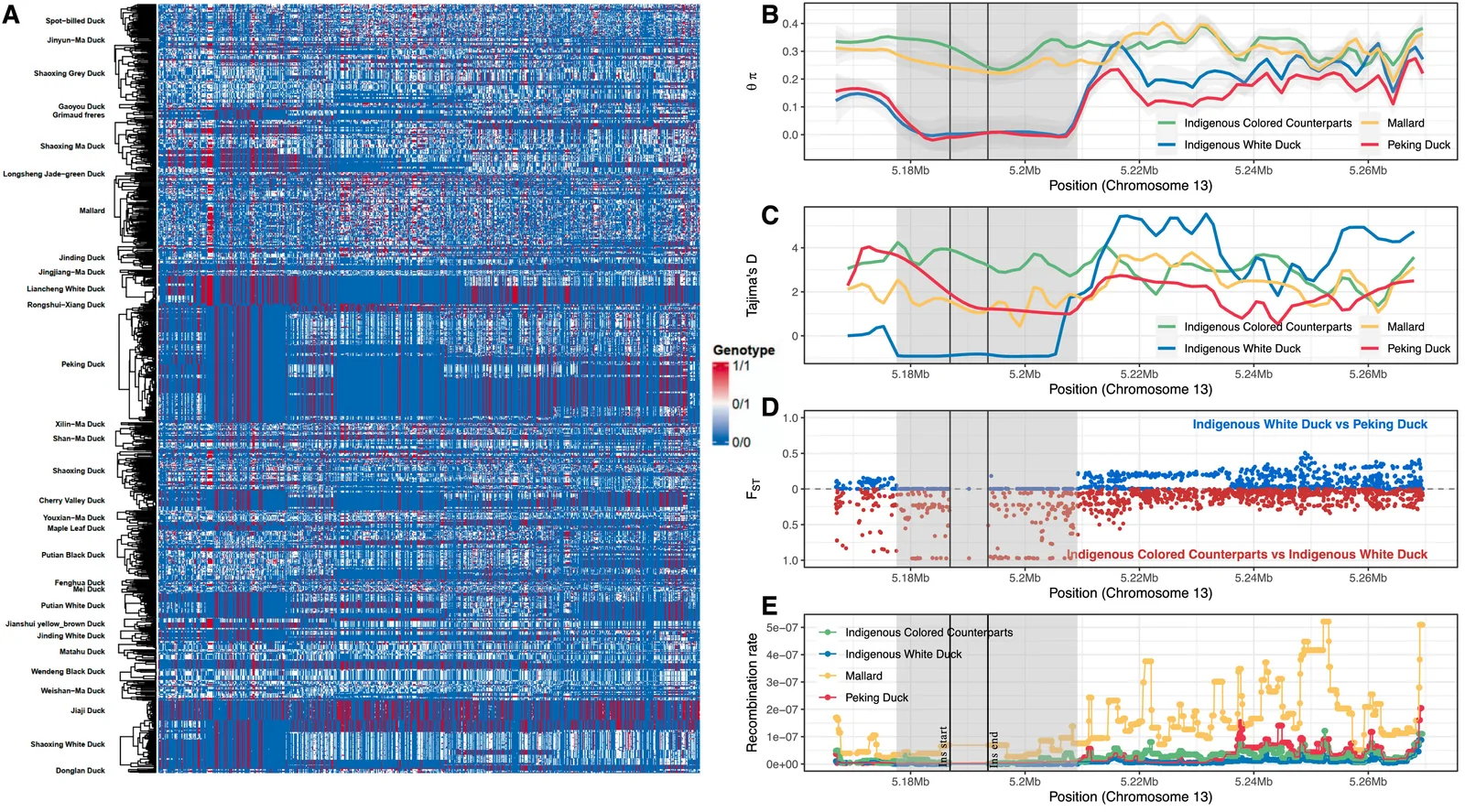
Genetic diversity and selection signatures at the *MITF* locus. (A) Genotype heatmap of *MITF* variants, with populations shown at breed level. (B) Nucleotide diversity (θπ). (C) Tajima's D. (D) Pairwise F_ST_ of Indigenous White Duck against Peking Duck (upper) and against Indigenous Colored Counterparts (lower, plotted downward from zero). (E) Estimated recombination rate. In (B) – (E), grey shading marks the ∼31.6 kb haplotype block and vertical lines the boundaries of the insertion. Indigenous White Duck comprises the white-plumage lines of Jinding, Putian black and Shaoxing ducks, and Indigenous Colored Counterparts the three populations from which these lines were derived, namely Jinding duck, Putian black duck and the grey-plumage line of Shaoxing duck. Per-line values of θπ and Tajima's D are shown in Fig. S3. Group assignments are listed in Table S3.

### Gene flow from commercial meat-type duck and the origin of the MITF ∼6.6 kb insertion in Chinese indigenous ducks

Population structure analysis established that the three indigenous white-plumage lines retain the genomic background of their respective colored-plumage counterparts rather than that of commercial meat-type ducks (Fig. 2). All three nonetheless carry the same *MITF* insertion and the same ∼31.6 kb haplotype block as commercial meat-type ducks (Fig. 3), prompting us to test directly for gene flow between the two.

D-statistics detected significant signals of gene flow from commercial meat-type ducks in all three indigenous white-plumage lines (|Z| > 3, Table S5). Signals were likewise detected in their colored-plumage counterparts, indicating that gene flow from commercial meat-type ducks is a general feature of these indigenous breeds rather than one acquired during development of the white-plumage lines.

We next used the f4-ratio to compare the extent of this gene flow between paired lines. Because the reference populations available are themselves not free of gene flow from commercial meat-type ducks, α was not interpreted as an absolute admixture proportion, and each white-plumage line was compared with its colored-plumage counterpart under identical (A, B, C, O) configurations. In all 32 configurations in which both lines yielded a significant estimate, α was higher in the white-plumage line, by 0.025 to 0.038 in Shaoxing duck, 0.019 to 0.031 in Jinding duck and 0.002 to 0.003 in Putian duck, consistent with a modest excess of commercial-derived ancestry retained alongside the insertion in the white-plumage lines (Fig. 4A).

**Fig. 4:**
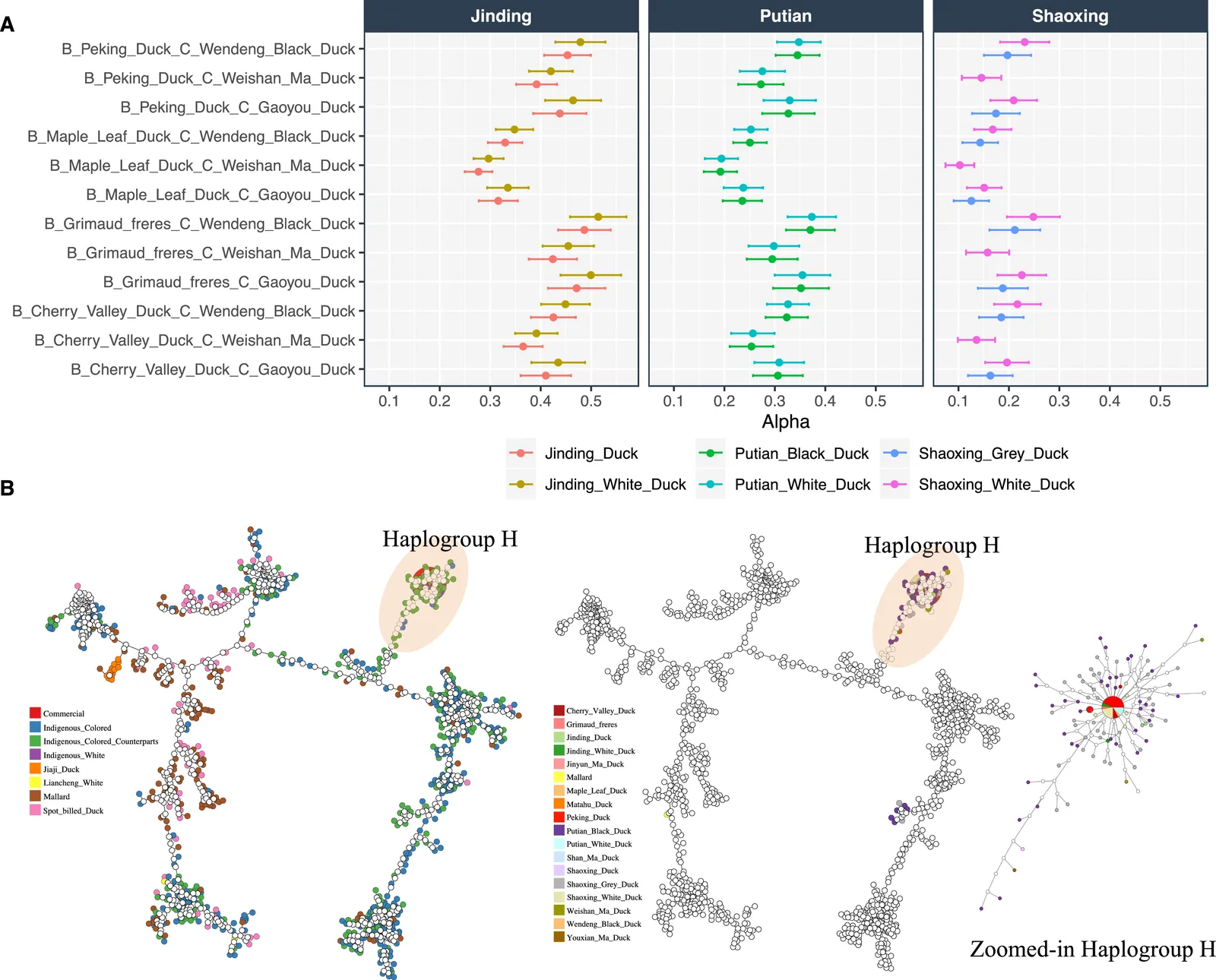
Origin analysis of the *MITF* insertion mutation in indigenous duck populations. (A) F4-ratio estimates (α), with Spot-billed Duck (*Anas poecilorhyncha*) as outgroup O, Mallard (*Anas platyrhynchos*) as population A, and the three indigenous white-plumage lines and their corresponding colored-plumage counterparts as test population X. In the y-axis labels, B and C denote source population B and reference population C, respectively. Only estimates with 0 < α < 1 and |Z| > 3 are shown. (B) Haplotype networks of the ∼31.6 kb block. Haplogroup H (highlighted) is the major branch composed of commercial meat-type ducks and indigenous white-plumage lines. Left, haplotypes colored by group (Table S3); open circles denote inferred haplotypes not observed in the samples. Middle, the same network with insertion-carrying haplotypes colored by breed. Right, haplogroup H enlarged, with the same coloring as the middle network. Node size is proportional to the number of haplotypes.

Haplotype-based analyses were then used to establish the origin of the insertion-carrying haplotypes themselves. The haplotype network of the ∼31.6 kb block contained a major branch, haplogroup H, composed of haplotypes from commercial meat-type ducks and indigenous white-plumage lines and containing no wild-duck haplotypes (Fig. 4B). Its core haplotype reached 78.4% in commercial meat-type ducks and 96.5% in indigenous white-plumage lines, versus only 1.6% in colored-plumage populations, and was absent from both Mallards and Spot-billed ducks. Commercial meat-type ducks and indigenous white-plumage lines shared this core haplotype together with its derivatives, forming a single tight cluster. A zoomed view of haplogroup H showed that the insertion-carrying haplotypes of indigenous colored-plumage populations occupy terminal positions derived from the core haplotype shared with commercial meat-type ducks, rather than basal positions within the haplogroup (Fig. 4B). Consistently, an NJ tree built from haplotype sequences of the block placed the insertion-carrying haplotypes of the indigenous white-plumage lines with those of commercial meat-type ducks and apart from their colored-plumage counterparts, in contrast to the NJ tree built from full chromosome 13 sequences (Fig. S5).

## Discussion

### The MITF ∼6.6 kb insertion as the common genetic basis for white plumage in indigenous duck

The genetic basis of white plumage in indigenous ducks has remained unclear despite extensive characterization of this trait in commercial meat-type ducks. In this study, GWAS combined with structural variant detection identified a ∼6.6 kb Gypsy insertion in the *MITF* gene as the most likely causative mutation underlying white plumage in the Shaoxing duck, and analysis of an expanded dataset spanning 38 populations confirmed that this insertion is the common genetic basis for white plumage across both commercial meat-type ducks and indigenous white-plumage lines, with the notable exception of Liancheng white duck, which relies on a distinct *PMEL*-based mechanism (Wang, et al., 2025). The transcriptional consequence of this insertion has been characterized: a pan-genome study by Wang et al. demonstrated that the Gypsy insertion between exon 1M and exon 2 disrupts MITF-M transcript expression and generates a novel transcript, thereby blocking the melanogenesis pathway (Wang, et al., 2024a); this is consistent with findings in mice showing that loss of the MITF-M isoform abolishes mature neural crest-derived melanocytes in the skin, producing a white coat phenotype (Flesher, et al., 2020). The *MITF* gene is driven by multiple promoters, giving rise to a variety of tissue-specific isoforms, of which MITF-M is the isoform specifically expressed in melanocytes (Shibahara, et al., 2000; Yasumoto, et al., 1998). The insertion falls precisely between the MITF-M specific exon (1M) and the downstream exon, selectively disrupting MITF-M transcription, which explains why individuals carrying the mutation exhibit only pigmentation loss, with no overt abnormalities in other tissue functions.

This mechanism carries particular implications for genetic research on plumage coloration in indigenous ducks. Because the insertion eliminates mature melanocytes, it is expected to exert a broad epistatic effect over downstream genes in the melanogenesis pathway: regardless of what variants occur in *MC1R* (Zhang, et al., 2023), *PMEL* (Wang, et al., 2025), or other genes, their effects cannot be manifested in the absence of mature melanocytes. This epistatic relationship suggests that, when conducting genetic studies of duck plumage coloration, the genotypic status of the *MITF* locus should be established as a priority.

### Historical inter-breed gene flow underlies the MITF insertion in Chinese indigenous egg-type white plumage duck

The *MITF* insertion shared between indigenous white-plumage lines and commercial meat-type ducks could in principle reflect recent hybridization during white-plumage line development. Population genetic structure analysis provides no support for admixture of this kind. Each white-plumage line clusters with its corresponding colored-plumage counterpart rather than with commercial meat-type ducks, and F3 statistics detected no evidence of large-scale admixture (Fig. 2, Table S4). This raises the question of how the same insertion came to be present across independently developed indigenous lines.

Four origins may be considered. The first is independent origin within indigenous populations. This can be excluded because the insertion arose approximately 0.6 million years ago (Wang, et al., 2024a), long predating duck domestication and even the divergence between mallard and Spot-billed duck (Zhang, et al., 2025). The second is direct inheritance from wild ancestors. This is likewise not supported. Only one heterozygous carrier was identified among 107 mallards and none among Spot-billed ducks (Fig. S2), and the dominant core haplotype of white-plumage populations is genetically distant from the insertion-carrying mallard haplotype (Fig. 4B). The third is vertical inheritance from a pool of birds common to the founding of both meat-type and egg-type lineages. Divergence of the two lineages at the time of founding, followed by independent accumulation of variation, would yield insertion-carrying haplotypes forming a group parallel to that of commercial meat-type ducks. Within haplogroup H, however, the insertion-carrying haplotypes of indigenous colored-plumage populations are nested within the commercial cluster and derived from its core haplotype (Fig. 4B). The fourth is introgression between breeds, and it is the explanation supported by the data. Insertion-carrying haplotypes from indigenous white-plumage lines cluster tightly with those of commercial meat-type ducks in both the haplotype network and the NJ tree (Fig. 4B, Fig. S5), and historical gene flow between commercial meat-type ducks and indigenous breeds has been documented previously (Zhang, et al., 2025).

Among the commercial populations examined, the four proved indistinguishable at this locus, each being fixed for the insertion (Fig. S2) and carrying the same ∼31.6 kb block (Fig. 3A), consistent with the derivation of Cherry Valley, Grimaud Frères and Maple Leaf ducks from Peking duck. The donor is therefore identified at the level of the Peking duck lineage rather than of any single present-day population. Insertion-carrying haplotypes outside haplogroup H are also present in the network (Fig. 4B), as expected for a variant of this age, but the haplotype fixed in the indigenous white-plumage lines is the one shared with commercial meat-type ducks. Low-frequency functional variants introduced by past inter-breed gene flow may thus serve as latent genetic resources readily mobilized under shifting selective pressures.

### Rapid fixation of a pre-existing haplotype block explains convergent sweep signatures in Chinese indigenous white plumage ducks

A striking observation in this study is that the indigenous white-plumage lines, despite their considerably shorter breeding histories, exhibit positive selection signals at the *MITF* locus nearly indistinguishable from those of commercial meat-type ducks, sharing an approximately 31.6 kb near-homozygous haplotype block. Two explanations may account for this pattern. First, only the insertion itself may have introgressed from Peking duck lineage into indigenous ducks, after which directional selection for white plumage, acting in combination with reduced recombination, independently generated a sweep of comparable extent in each breed. Second, the block already established in Peking duck lineage may have been introgressed as a unit, with subsequent selection acting on this pre-existing block rather than generating a sweep de novo.

The two explanations differ in the haplotype expected to be fixed in each line. Under the first, selection would have acted on whatever insertion-carrying haplotypes were present in each indigenous population, and the three lines, developed separately and from different breeds, would not be expected to converge on one of them. Under the second, all three would carry the haplotype that predominates in the donor lineage. Each of the three lines is fixed for the same core haplotype, the one that predominates in commercial meat-type ducks, and the additional haplotypes they carry are derived from it (Fig. 4B), supporting the second explanation. Consistent with this, the derived haplotypes differ from the core by one or a few mutations, and Tajima's D within the block is negative in the indigenous white-plumage lines (Fig. 3C), particularly in the Jinding and Shaoxing lines (Fig. S3B), as expected when variation has accumulated on the introduced haplotype since its transfer.

The integrity of the block across populations follows from the properties of the region. The insertion arose only once (Wang, et al., 2024a) and lies in an interval that recombines at a markedly reduced rate in Mallard and in the indigenous colored-plumage populations as well as in the white-plumage lines (Fig. 3E), so an introgressed segment carrying it would have spanned the block and would have been eroded only slowly thereafter. Fixation was correspondingly rapid. The recessive inheritance of the insertion means that any bird displaying white plumage is homozygous, so that once white-plumage individuals appeared they could be used directly as parents and the allele reached fixation without the intervening generations of selection on carriers that a dominant variant would require. The combination of suppressed recombination and rapid fixation under recessive inheritance thus accounts for the preservation of the block after its introduction: the haplotype that was already homogeneous in the Peking duck lineage was fixed before recombination could erode it, producing sweep signatures in the indigenous egg-type white-plumage lines nearly indistinguishable from those in commercial meat-type ducks.

## Conclusions

Through GWAS combined with structural variant detection, this study identified a ∼6.6 kb Gypsy insertion in *MITF* as the most likely causative mutation underlying white plumage in Shaoxing ducks, and validated through an expanded dataset that this mutation represents the common genetic basis for white plumage across commercial meat-type and egg-type ducks, with the exception of Liancheng White duck. The development of indigenous egg-type white-plumage lines did not involve hybridization with commercial meat-type ducks; rather, the insertion mutation was introduced through historical gene flow from the Peking duck lineage, carried on a haplotype block already homogeneous in that lineage, persisted at low frequency over an extended period, and was subsequently driven to rapid fixation under recent directional selection. These findings illustrate a broader evolutionary dynamic in which low-frequency functional variants introduced via historical inter-breed introgression can respond rapidly to shifting selective pressures and reach fixation within a short timeframe, generating selective sweep signatures nearly indistinguishable from those produced by prolonged selection.

## Author contributions

Chunyuan Zhang, Lizhi Lu, and Li Chen conceived and designed the study, with additional input from Chungang Feng and Yiqiang Zhao. Chunyuan Zhang performed the analyses, prepared the figures, and drafted the manuscript. Zhen Chen contributed to the data analyses and figure preparation, with additional assistance from Pan Li and Panxue Wu. Xuge Wang performed the cross-breeding experiments and collected the samples. Chungang Feng, Yiqiang Zhao, Li Chen and Lizhi Lu contributed to the interpretation of the results, revised the manuscript and supervised the work. Li Chen acquired the funding and supervised the overall study. All authors read and approved the final version of the manuscript and agree to be accountable for all aspects of the work.

## Disclosures

The authors declare that they have no known competing financial interests or personal relationships that could have appeared to influence the work reported in this paper.

## Data Availability

The raw whole genome sequencing data generated in this study have been deposited in the NCBI Sequence Read Archive (SRA) under accession number PRJNA1433051 (https://www.ncbi.nlm.nih.gov/sra/?term=PRJNA1433051)

## Footnotes

Supplementary material associated with this article can be found, in the online version, at doi:10.1016/j.psj.2026.107827.
