# Supplementary material for: Historical introgression from commercial meat-type ducks constitutes the common genetic basis of white plumage in egg-type duck breeds
Source: Poult Sci. 2026 Sep 22;105(12):107827. doi: 10.1016/j.psj.2026.107827 (PMC13677777; doi:10.1016/j.psj.2026.107827)
Supplement: Supplementary file 1 [file mmc1.docx]

**Figure S1 Schematic diagram of the ~6.6 kb Gypsy insertion in *MITF*.** The element lies in the orientation opposite to the plus strand of the reference assembly and is therefore drawn with the 3′ long terminal repeat (LTR) on the left. The two LTRs (1,287 bp each) flank a 4,053 bp internal region, and the element is bordered by a 5-bp target site duplication (TSD). TG/CA, terminal dinucleotides of the LTRs; PPT, polypurine tract; pos 1014, the site at which the two LTRs differ.

**Figure S2. Frequency of the *MITF* ~6.6 kb insertion across populations.** Bars show genotype counts, and values above the bars give the insertion allele frequency. Ins/Ins, Ins/Del and Del/Del correspond to genotypes 0/0, 0/1 and 1/1, respectively. Shaoxing Duck is from public data; Shaoxing Ma Duck, Shaoxing Grey Duck and Shaoxing White Duck were sequenced in this study. Shaoxing Grey Duck and Putian Black Duck were sampled from cross populations.

**Figure S3. Nucleotide diversity and Tajima's D statistic for each population across the *MITF* locus.** (A) Nucleotide diversity (θπ) with 95% confidence intervals shown as shaded ribbons. (B) Tajima's D. The grey box marks the ~31.6 kb haplotype block, and vertical lines mark the boundaries of the insertion.

**Figure S4. Extended haplotype homozygosity (EHH) analysis surrounding the *MITF* insertion mutation site.**

**Figure S5. Neighbor-joining (NJ) phylogenetic trees constructed from haplotype sequences.** (A) NJ tree based on chromosome 13 haplotype sequences of all samples. (B) NJ tree based on ~31.6 kb block haplotype sequences of all samples. Tips are colored by group (Table S3).

**Figure S6. Pairwise F_ST_ among the three white-plumage lines.**
